# The epidemiological and clinical characteristics of Langerhans cell sarcoma in the United States: A population study based on SEER data from 2000 to 2019

**DOI:** 10.1097/MD.0000000000039315

**Published:** 2024-08-16

**Authors:** Bahaa Mali, Ali Mali, Alaa Mali, Mohammed Abdulrazzak, Afnan W. M. Jobran

**Affiliations:** a Faculty of Medicine, Al Quds University, Jerusalem, Palestine; b Faculty of Medicine, University of Aleppo, Aleppo, Syria.

**Keywords:** Langerhans cell histiocytosis, Langerhans cell sarcoma, rare diseases, SEER, survival rate

## Abstract

Langerhans cell sarcoma (LCS) is a rare aggressive malignancy with a poor prognosis. Our knowledge about this condition is limited and mainly based on case reports, making it challenging to understand its epidemiology, clinical features, and patient outcomes. We conducted a retrospective study of LCS patients diagnosed between 2000 and 2019 using the Surveillance, Epidemiology, and End Results (SEER) database. The data were stratified based on age, race, stage, clinical pattern, and treatment method. Our study found that 57 LCS cases were reported in SEER registries between 2000 and 2019. Among these cases, most patients (50.9%) were over 60 years old and White (71.9%) with almost equal males to females ratio. About 45.6% of cases were localized while 47.4% were at distant stages. Of the patients, 50.9% underwent surgery, 45.6% received chemotherapy, and only 21.1% received radiotherapy. The overall survival rate for patients diagnosed with LCS in the United States is generally low with a 1-year overall rate of 63.8%. Certain factors can negatively impact prognosis, such as advanced stages of the disease, secondary tumors, or more than 1 tumor per patient. LCS is a rare disease with poor survival rates. Future research should incorporate global data for further statistically significant results. Moreover, investigating the molecular, genetic, and pathophysiological backgrounds of these tumors is crucial for developing targeted management strategies and improving prognosis.

## 1. Introduction

Langerhans cells are a specific type of dendritic cells that reside in the epidermis. They function as professional antigen-presenting DCs, playing a crucial role in the innate and adaptive immune systems.^[1]^ Langerhans cell sarcoma (LCS) and Langerhans cell histiocytosis (LCH) are proliferative conditions that involve cells that exhibit Langerhans cell features. While LCH is a clinically benign condition, LCS is a rare and malignant tumor that shows greater cytological atypia and clinical aggressiveness.^[2]^

Due to its rare occurrence, LCS is not well understood. Our knowledge about this condition mainly relies on case reports, leading to a limited understanding of its epidemiology, clinical features, patient outcomes, and lack of evidence-based treatment strategies.^[3]^ To our knowledge, one registry-based study investigated this disease between 2001 and 2014 and found an overall incidence rate of 0.2 per 10,000,000 without significant differences by sex or race. The study primarily relied on the National Cancer Data Base (NCDB) and, to a lesser extent, the Surveillance, Epidemiology, and End Results (SEER) database.^[4]^

To enhance our understanding of LCS, we analyzed the SEER database to investigate patient demographics, clinical characteristics, presentation sites, and survival outcomes among patients diagnosed between 2000 and 2019.

## 2. Methods

We conducted this retrospective study on patients diagnosed with LCS between 2000 and 2019 using the SEER*Stat software (version 8.3.9.2 developed by the National Cancer Institute, Bethesda, Maryland). To ensure maximum coverage of SEER geographic regions, we used SEER 22 registries for patients diagnosed in 2006 and afterward.^[5]^ However, for patients diagnosed before 2006, we used SEER 17 registries since SEER 22 had incomplete stage and treatment data for that period.^[6]^ This study was based on anonymized data extracted from the publicly available SEER registry. Hence, institutional review board approval was not required. In conducting this retrospective study, we adhered to the Strengthening the Reporting of Observational Studies in Epidemiology (STROBE) Guideline.^[7]^

We identified cases of LCS that were confirmed histologically using the International Classification of Diseases for Oncology, 3rd edition, histology code 9756/3. We used descriptive statistics to summarize the epidemiological and clinical features of our cohort using Jamovi software. We extracted and analyzed data on age, sex, race, year of diagnosis, total number of tumors per patient, first primary tumor (yes or no), primary site, treatment (surgery, radiotherapy, and chemotherapy), survival time, and survival status. Cases of LCS were categorized into 9 categories based on the primary site of involvement: connective tissue, lung, bones and joints, mouth or external ear, pancreas, reticuloendothelial system, skin, urinary system, and unknown.

We calculated overall survival rates for 6 months and 1 year and compared the differences between the various epidemiological, clinical, and treatment subgroups using log-rank analysis. We performed survival analysis with R statistical software Version 4.3.2 and defined statistical significance as *P* < 0.05.

## 3. Results

A total of 57 cases of LCS were identified from the SEER program between 2000 and 2019. Among these cases, 50 were reported in the SEER 22 registries since 2006, while 7 were reported in the SEER 17 registries before 2006. The majority of patients (50.9%) were over 60 years old, White (71.9%), and diagnosed between 2011 and 2019 (63.2%). The gender distribution was almost equal, with 50.9% males and 49.1% females. Approximately 45.6% of cases were localized at the time of diagnosis, while 47.4% were at distant stages (Table 1).

**Table 1:** Baseline characteristics of patients diagnosed with Langerhans cell sarcoma (LCS) from 2000 to 2019 based on SEER database.

Variable	Count	n (%)
Total	57	100%
Age group (yr)		
<30	13	22.8%
30–60	15	26.3%
>60	29	50.9%
Sex		
Male	29	50.9%
Female	28	49.1%
Year of diagnosis		
2002–2010	21	36.8%
2011–2019	36	63.2%
Race		
NHW	41	71.9%
NHB	6	10.5%
NHAPI	3	5.3%
Hispanic	7	12.3%
Stage		
Localized	26	45.6%
Distant	27	47.4%
Unstaged	4	7%
First primary tumor		
Yes	38	66.7%
No	19	33.3%
Number of tumors		
1	35	61.4%
2 or more	22	38.6%
Surgery		
Yes	29	50.9%
No/unknown	28	49.1%
Chemotherapy		
Yes	26	45.6%
No/unknown	31	54.4%
Radiation		
Yes	12	21.1%
No/unknown	45	78.9%

NHAPI **=** Non-Hispanic Asian or Pacific Islander, NHB = Non-Hispanic Black, NHW **=** Non-Hispanic White.

The most common primary sites of LCS were connective tissue (36.8%), reticuloendothelial system (28.1%), bones and joints (14.0%), and skin (8.8%). The majority of patients (66.7%) had LCS as their first primary tumor, and 61.4% had only one total number of tumors. In terms of treatment, 50.9% of patients underwent surgery, 45.6% received chemotherapy, and a small proportion (21.1%) underwent radiotherapy (Table 2).

**Table 2:** Counts and percentages of patients diagnosed with Langerhans cell sarcoma (LCS) from 2000 to 2019 based on the primary site of presentation in the SEER database.

Primary site	Counts	n (%)
Connective tissue	21	36.8 %
Lung	1	1.8 %
Bones and joints	8	14.0 %
Mouth or external ear	2	3.5 %
Pancreas	1	1.8 %
Reticuloendothelial system	16	28.1 %
Skin	5	8.8 %
Urinary system	1	1.8 %
Unknown	2	3.5 %

The overall survival rates for patients diagnosed with LCS in the United States were generally low. At 6 months, the survival rate was 76.8%, and at 1 year, it was 63.8%. The 1-year survival rates varied across different age groups, with patients under the age of 30 having a rate of 69.2%, those between the ages of 30 and 60 having a rate of 60%, and those over the age of 60 having a rate of 64.3%. These differences were not statistically significant (*P* > .05). Similarly, the 1-year survival rates did not differ significantly among racial groups, with non-Hispanic Whites having a rate of 70.1%, non-Hispanic Blacks having a rate of 33.3%, non-Hispanic Asians or Pacific Islanders having a rate of 66.7%, and Hispanics having a rate of 50% (*P* > .05) (Table 3).

**Table 3:** Six-month and 1-year survival rates based on Kaplan–Meier analysis, subgrouped by different variables.

Variable	Survival % (95% CI) at 6 months	Survival % (95% CI) at 1 year
Total	76.8% (66.5–88.7)	63.8 (52.3–77.9)
Age group (yr)		
<30	69.2% (48.2–99.5)	69.2% (48.2–99.5)
30–60	86.7% (71.1–100%)	60% (39.7–90.7)
>60	75 (60.6–92.9)	64.3 (48.8–84.7)
Race		
NHW	85.4 (75.2–96.9)	70.1 (57.2–85.8)
NHB	50 (22.5–100)	33.3 (10.8–100)
NHAPI	66.7 (30–100)	66.7 (30–100)
Hispanic	50 (22.5–100)	50 (22.5–100)
Stage		
Localized	88.5 (77–100)	84.4 (71.5–99.7)
Distant	65.4 (49.4–86.5)	46.2 (30.5–69.9)
Unstaged	75 (42.6–100)	50 (18.8–100)
First primary tumor		
Yes	81.6 (70.1–94.9)	70.4 (57.1–86.8)
No	66.7 (48.1–92.4)	50 (31.5–79.4)
Number of tumors		
1	82.9 (71.3–96.3)	73.8 (60.4–90.1)
2 or more	66.7 (49.3–90.2)	47.6 (30.4–74.6)
Surgery		
Yes	86.2 (74.5–99.7)	68.5 (53.4–87.9)
No/unknown	66.7 (51.1–87)	58.8 (42.8–80.9)
Chemotherapy		
Yes	76.9 (62.3–94.9)	60.2 (43.7–82.9)
No/unknown	76.7 (62.9–93.4)	66.7 (51.8–85.9)
Radiation		
Yes	75 (54.1–100)	66.7 (44.7–99.5)
No/unknown	77.3 (65.8–90.7)	63 (50.1–79.2)

NHAPI **=** Non-Hispanic Asian or Pacific Islander, NHB **=** Non-Hispanic Black, NHW **=** Non-Hispanic White.

The stage of LCS had a significant impact on overall survival rates. Patients diagnosed with distant-stage cancer had the lowest 6-month and 1-year survival rates (65.4% and 46.2%, respectively), while those diagnosed with localized-stage cancer had the highest 6-month and 1-year survival rates (88.5% and 84.4%, respectively) (*P* = .023, log-rank test). Additionally, patients with the first primary tumor and those with only one total number of tumors per patient had better survival outcomes compared to those with a secondary tumor or with 2 or more total tumors per patient (*P* = .017, *P* = .014 log-rank test, respectively) (Table 3).

Among the treatment modalities, patients who underwent surgery had a higher 1-year survival rate (68.5%) compared to those who did not undergo surgery or whose status was unknown (58.8%). Patients who received radiotherapy also had higher survival rates (66.7%) compared to those who did not receive radiotherapy or whose status was unknown (63%). However, chemotherapy did not show an improvement in overall survival, and none of the treatment modalities showed a statistically significant impact on the log-rank test (*P* > .05).

## 4. Discussion

Our analysis revealed that LCS is an extremely rare malignancy, with only 57 reported cases to the SEER registry in the United States over 19 years (2000–2019). The study participants were divided into 3 age groups for the purpose of clinically relevant comparisons: under 30 years, 30–60 years, and over 60 years. These age groupings were selected based on commonly used cutoffs in oncology research to enable meaningful comparisons across different life stages. This method was employed to facilitate a comprehensive analysis of age-related effects on treatment outcomes and survival rates.

Among all cases, the majority of patients (50.9%) were over 60 years old, White (71.9%), and diagnosed between 2011 and 2019 (63.2%). These results are consistent with a previous registry-based study that showed an incidence rate of 0.2 per 10,000,000 without any significant differences by sex or race. Using the NCDB, they found 52 patients with LCS between 2004 and 2015. The median age at diagnosis was 62. However, no cases were reported in the Hispanic population, and 60% of the cohort were males.^[4]^ In our cohort, 7 patients (12.3%) were of Hispanic race, and the percentage of males (50.9%) and females (49.1%) was almost equal.

In our cohort, 45.6% of cases were found to be localized, and 66.7% of patients had LCS as their first primary tumor. Meanwhile, 47.4% of cases were at a distant stage, and 38.6% of patients had 2 or more tumors. A previously published review of all reported LCS cases showed that 40.9% of cases were advanced, 33.3% had single-site involvement, and 25.8% had locoregional involvement.^[3]^

In terms of treatment, surgery was performed on 50.9% of the patients, while 45.6% underwent chemotherapy. Only a small proportion of the patients (21.1%) received radiotherapy. Due to the rarity of this malignancy, there is a limited understanding of LCS pathophysiology, and there is a lack of evidence regarding the most appropriate treatment. Most treatment protocols are based on empirical evidence and have shown limited success.^[3,4,8,9]^

Our analysis of survival rates indicates that LCS generally has a poor prognosis. The overall survival rate after 6 months is 76.8%, and it drops to 63.8% after 1 year. Age and race did not significantly impact overall survival based on the log-rank test. Patients with localized, first primary tumors and those with only one total number of tumors per patient had higher survival rates compared to patients diagnosed with distant-stage cancer, secondary tumors, or with 2 or more total tumors per patient. Similarly, the published review of the previously reported cases of LCS revealed a significant difference in both disease-specific survival (DSS) and disease-free survival between local, locoregional, and disseminated disease cohorts. Specifically, patients with local disease had a 5-year DSS rate of 70% (SE, ±12.8). Patients with locoregional disease had a 5-year DSS rate of 15% (SE,±9.5), while none of the patients with disseminated disease survived for 5 years.^[3]^

In our study, no statistically significant difference was observed with any treatment modality, including surgery, chemotherapy, or radiotherapy (*P* > 0.05 for all). Nevertheless, none of these treatment modalities reached a statistically significant impact on survival. This could be due to the rarity of the disease, resulting in smaller sample sizes in each treatment subgroup. The small sample size might have influenced the statistical analysis, making it difficult to draw meaningful conclusions.

Several studies have suggested that surgical removal with clear margins can improve the survival rate of patients with localized disease.^[3,10–12]^ Yoshimi et al have stated that surgery has a significant role in treating limited diseases. However, the role of adjuvant therapy in such cases remains uncertain. They found that patients who had a complete or partial response had undergone adjuvant therapy either before or at the time of the initial surgery. In cases where adjuvant therapy was delayed, the outcomes were worse.^[8]^ However, a sregistry-based study among LCS patients in the United States has demonstrated that surgical therapy did not lead to a statistically significant survival advantage, even after excluding patients with primary bone marrow and reticuloendothelial disease. The results can be attributed to the small sample size of the treatment subgroups, which could be due to the rarity of the disease.^[4]^

While radiotherapy has been useful in treating the less severe counterpart of the condition, LCH, its effectiveness in treating LCS remains unclear.^[13]^ Few cases of complete responses to localized disease have been observed.^[14,15]^ However, similar promising outcomes have not been observed in disseminated diseases.^[16]^ Tella et al found that radiotherapy improved overall survival rates in patients with LCS primary sites other than bone marrow or reticuloendothelial system, based on the NCDB data.^[4]^

Tella et al reported that chemotherapy did not substantially increase the overall survival rate of patients with systemic disease and involvement of the bone marrow and the reticuloendothelial system.^[4]^ Previous case reports have demonstrated that the response to chemotherapeutic drugs, either in combination or alone, can vary. This variation in response may be attributed to the aggressiveness of the disease and our insufficient understanding of its precise pathophysiology, leading to the empirical use of various hematological and sarcoma regimens, such as the ESHAP regimen (etoposide, carboplatin, cytarabine, and methylprednisolone) and the CHOP regimen (cyclophosphamide, doxorubicin, vincristine, and prednisone).^[3,8,17]^

The trends observed in our study, although not statistically significant, might suggest that the aggressiveness of LCS makes it challenging to assess the impact of treatments. The rarity of the disease results in small sample sizes, limiting the statistical power of our analyses. Longer-term survival metrics, such as 3- or 5-year overall survival and cause-specific survival, could provide additional insights into the long-term effects of treatment modalities. Future research should focus on these longer-term outcomes to better understand treatment efficacy.

Our study has some limitations. As this was an analysis based on population-level data, we did not have access to detailed patient information, such as comorbidities and healthcare access, which may have influenced the survival rate of the patients. Additionally, there may be a bias toward health trends among patients in the United States compared to worldwide trends, and there is a lag between current treatment methods for diseases and those recorded in the SEER database. This retrospective cohort design may have resulted in some bias in our findings. However, the SEER database’s high-quality registry provides reliable data, especially valuable for studying the epidemiological and clinical features of rare malignancies.

## 5. Conclusion

LCS is a rare disease with a poor prognosis. The stage of the disease has an important role in determining overall survival rates. Patients with secondary tumors or more than one tumor often have a substantially poorer prognosis. Although treatment with radiotherapy and surgery has shown a modest increase in survival rates, this improvement is not statistically significant, possibly because of the small sample size in each treatment group. Because this condition is exceedingly uncommon, collecting statistically meaningful data has been challenging. Future research that incorporates data from various cancer registries throughout the world will be required to produce more meaningful results. It is also critical to investigate the molecular, genetic, and pathophysiological backgrounds of these tumors to create more targeted management strategies that can improve prognosis.

## Author contributions

**Conceptualization:** Bahaa Mali, Ali Mali, Alaa Mali, Mohammed Abdulrazzak

**Data curation:** Bahaa Mali, Ali Mali, Alaa Mali

**Investigation:** Bahaa Mali, Ali Mali

**Methodology:** Bahaa Mali, Ali Mali

**Project administration:** Bahaa Mali, Ali Mali

**Resources:** Bahaa Mali, Ali Mali, Alaa Mali, Mohammed Abdulrazzak

**Validation:** Bahaa Mali, Ali Mali, Alaa Mali, Mohammed Abdulrazzak, Afnan W. M. Jobran

**Writing – original draft:** Bahaa Mali, Ali Mali, Alaa Mali, Mohammed Abdulrazzak, Afnan W. M. Jobran

**Writing – review & editing:** Bahaa Mali, Ali Mali, Alaa Mali, Mohammed Abdulrazzak, Afnan W. M. Jobran

**Funding acquisition:** Ali Mali, Alaa Mali

**Supervision:** Mohammed Abdulrazzak, Afnan W. M. Jobran
